# A qualitative study of the experiences of young people with severe mental health problems and complex needs regarding youth flexible assertive community treatment

**DOI:** 10.3389/fpsyt.2024.1478345

**Published:** 2024-12-06

**Authors:** Marthe Johansen, Hanne Kilen Stuen, Eva Brekke, Camilla Bergsve Jensen, Anne Landheim

**Affiliations:** ^1^ Department of Health and Nursing Sciences, Inland Norway University of Applied Sciences, Elverum, Norway; ^2^ Norwegian National Advisory Unit on Concurrent Substance Abuse and Mental Health Disorders, Innlandet Hospital Trust, Hamar, Norway

**Keywords:** qualitative study, young people, severe mental health problems, youth flexible assertive community treatment, child and adolescent mental health services, youth-friendly mental health services

## Abstract

Youth Flexible Assertive Community Treatment (Youth Flexible ACT) is a service model for children and young people with severe mental health problems and complex needs aimed at providing integrated, continuous and holistic care. Studies on young people’s experiences of Youth Flexible ACT or similar models are scarce. The present qualitative study aimed to explore and describe how young people with severe mental health problems and complex needs experience follow-up and treatment provided by Youth Flexible ACT teams. Semi-structured interviews were conducted with 14 young people (age range, 15–19 years) who were being followed up by a Youth Flexible ACT team. Qualitative content analysis was used, and the following two overarching themes characterizing the young people’s experiences of follow-up and treatment provided by Youth Flexible ACT teams were identified: (1) trusting and collaborative relationships, and (2) organization matters. The participants experienced a more personal relationship with the staff, who behaved more like friends and paid attention to resources, interests, solutions and their context. The participants emphasized and valued components that coincided with the Youth Flexible ACT model, indicating a match between what the young people needed and wanted and what the model was supposed to provide. The team providing these relationships and youth-friendly and developmentally sensitive care appeared to be facilitated by how the service model was organized, with the teams being flexible and accessible while providing integrated, multifaceted help and systemic follow-up.

## 1 Introduction

Mental health issues are one of the major challenges in the field of public health, especially for young people (1). As many as 50% of young people are said to be affected by mental disorders by the age of 25 years (2, 3). These problems are increasing in young people (2, 4, 5), and include anxiety, depressive symptoms, psychological distress and suicide (6–8). McGorry et al. (2) emphasized the urgent seriousness of this issue as follows: ‘*The rising tide of mental ill-health in young people globally demands that this focus be elevated to a top priority in global health*’.

A group of young people struggle with more severe mental health problems and have complex needs, leading to serious limitations in psychosocial functioning. These young people are difficult to engage in mainstream mental health services (9–11). Challenges in children and adolescent mental health services (2, 12, 13) have been well documented in several reports, especially for the described group of vulnerable children and youth (9). Such challenges include complex and fragmented services (14). In addition, both primary and specialized mental health care are described as poorly designed and having problems meeting the youth population’s cultural and age-related developmental needs (2, 15). Health services lack the competence and framework required to provide adequate treatment, specifically for the group of young people with concurrent substance abuse and mental health issues (16, 17). Engaging youth with complex needs in existing services, in addition to premature termination from treatment, is considered a major challenge (18, 19).

The improvement of mental health services for children and young people worldwide (2, 20) and the development of integrated and youth-friendly health services (21, 22) are needed. Youth-friendly services are defined by the World Health Organization as accessible, acceptable and appropriate to young people (23). Multidisciplinary teams working seamlessly across all sectors need to overcome these challenges (10, 20).

Many approaches have been implemented for integrated youth mental health services (14, 24), but no consensus has been reached regarding the best practice (21). Examples of such services include Jigsaw in Ireland (25), Youthspace in England and Headspace in Australia (24, 26). These services share a holistic and integrated approach, working in multiple areas such as school and family, and with youth-engagement as a core component. They also focus on preventing severe mental health problems. However, the target group for these services is not specifically young people with the most severe difficulties, Youth Assertive Community Treatment (Youth ACT) and Early Intervention in Psychosis teams have been set up for those with the most severe problems and intensive care needs. Youth ACT is a team-based community approach with key features being the provision of outreach and holistic support, were young people with psychotic disorders are the primary target group (27). A recent review investigated service models for young people (age range, 12–25 years) with severe and complex mental illness. Services recommended for this population included intensive community treatment services or integrated inpatient services of short duration (less than one month) and community treatment using effective, established models (e.g. assertive outreach or multisystemic therapy). No service model alone was sufficient to meet the needs of this target group and the authors concluded that there was limited information describing models for the target group (10).

Youth Flexible Assertive Community Treatment (Youth Flexible ACT) has been set up and deployed widely throughout the Netherlands (28) to meet the described challenges. It is also currently being piloted in Sweden and Denmark. Norwegian health authorities have chosen to pilot and implement Youth Flexible ACT to achieve integrated and multidisciplinary care, and teams are becoming more widespread in Norway, with approximately 24 teams active as of June 2024. Youth Flexible ACT is an adapted variant of the adult Flexible ACT model (29) and represents both a new way to organize services and a new treatment model. In Norway, Youth Flexible ACT is a binding type of collaboration between primary mental health care and specialist services, integrating two care levels in one team. Youth Flexible ACT teams are multidisciplinary and assertive outreach teams for children and youth (age range, 12–25 years) with severe mental health problems, possible substance use and complex service needs (30). There is no requirement that the young person must fulfil diagnostic criteria to be enrolled in Youth Flexible ACT. Upon enrolment, less emphasis is placed on diagnoses, and more on functioning. Youth Flexible ACT teams are encouraged to provide as many services as possible and if needed, the team cooperates and coordinates with other services. The core principles of the Youth Flexible ACT model are flexibility, assertive outreach and a multidisciplinary team approach to stimulate participation in the local community, family and systemic work and recovery and user involvement, ensure follow-up in a continuous, holistic and integrated manner and use evidence-based methods and practices (31).

Studies on Youth Flexible ACT are scarce, both internationally and in Norway (28, 32, 33), and to our knowledge, only one qualitative study has been conducted (34). That case study found the Youth Flexible ACT model to be tailored to the needs of young people, integrated and flexible, thereby enabling young people to be engaged in care (34). In addition, few studies have investigated the Youth ACT model (27, 35) and there are relatively few studies on young people’s experiences with mental health services (36, 37), especially from the perspective of young people with severe mental health problems (9, 38, 39). In addition, feedback from service users is often collected and presented quantitatively (40, 41). For services to meet the needs of this group more successfully, be developmentally sensitive and youth-friendly and promote positive development, additional knowledge is needed about the experiences of children and young people, voiced by themselves (42–44).

Given this background, the present study aimed to explore and describe how young people with severe mental health problems and complex needs experience follow-up and treatment as provided by Youth Flexible ACT teams.

## 2 Materials and methods

### 2.1 Design

A qualitative study with a descriptive and exploratory design was selected because experiences with Youth Flexible ACT is an understudied area. The present study is based on a phenomenological hermeneutic epistemology that aims to understand phenomena based on the young people`s perspectives and everyday lived lives, but also with the understanding that all meaning are structured by an unconscious preunderstanding within a specific culture and historical era, where we encounter new phenomena with our previous horizon of understanding (45). We collaborated with a working group consisting of Youth Flexible ACT team members, one member from the Norwegian Directorate of Health, the county governor of Innlandet, the first and last author and two members with lived experiences of mental health problems at a young age as well as service user experience. In particular, the input of the members with lived experiences made the guide more relevant and youth-friendly (46). The working group provided advice and feedback regarding study planning, recruitment strategies, the development of interview guides and the interpretation of the results.

### 2.2 Setting

The study involved the first three Youth Flexible ACT teams in Norway, which started in the spring/summer of 2020. The teams were organized as a binding collaboration between primary mental health care and specialist services. The teams were multidisciplinary, comprising a psychologist, nurse and peer specialist. Two teams had a social worker, a doctor and a mercantile. Several providers were part-time employees in the Youth Flexible ACT team and simultaneously employed in other municipal or specialist services while working on assertive outreach. As of May 2022, a total of 55 young individuals were included in the three teams. Team members reported having a low threshold for admitting young people. Furthermore, they reported having some young people with suspected or diagnosed psychosis issues and other serious disorders, but fewer than they had expected with the most severe conditions. The characteristics of the young individuals included in the three teams indicated that they had significant and complex problems.

### 2.3 Recruitment

Convenience sampling was used with aim of recruiting participants who could provide experiences of treatment from a Youth Flexible ACT team. The inclusion criteria were 1 participants who 1) spoke Norwegian fluently enough to have no need of an interpreter and 2) currently received support from the Youth Flexible ACT team with a duration of at least six months. Considering that these young people often had severe mental health problems and difficulty in taking initiatives, and might thus be difficult to recruit, we stipulated no other inclusion criteria. Therefore, we instructed the team staff to provide the invitation to participate to all youth, regardless of the severity of their symptoms and illness.

Staff from the Youth Flexible ACT team, preferably the one(s) who knew the young person best, provided information about the study. We wrote a study information sheet about the study for the young people, which the staff handed out. Those who were interested in participating were informed that they could have a talk with the interviewer to ask questions before the interview. Since these are young people with various difficulties that might affect their ability to participate in research, such as challenges with initiative and attendance, we adopted a flexible approach in the recruitment process. This flexibility involved the following points: 1) Those who wanted to participate signed a written consent and gave it to the staff or to the interviewer. The staff coordinated the recruitment process and interview appointments were made in consultation with the first author. 2) Some appointments were made by the staff, and some were made by phone between the interviewer and the young person. 3) We made repeated appointments with several of them to facilitate their participation.

### 2.4 Participants

Five boys and nine girls (mean age, 16 years; age range, 15–19 years) participated in this study. All participants and parents of the participant under 16 years, signed an informed consent. The majority of the participants said that they wanted to receive follow-up by the Youth Flexible ACT team, except for one who did not want it at the time of referral but was positive about it at the time of the interview. All participants reported having psychological problems and/or diagnoses, including depression, behaviour difficulties, psychotic problems, childhood trauma, anxiety, high-functioning autism, post-traumatic stress disorder, eating disorder and personality disorder. All participants reported school problems ranging from smaller adaptions to being absent from school for the last 2–3 years. One participant reported problems with substance use. Some talked about family conflict as the reason for follow-up by the Youth Flexible ACT team. We did not collect data about diagnoses from medical records. However, most participants reported experiencing or having experienced multiple problems in their lives, (psychological, functional, and social). Eleven of the 14 participants had former treatment experience from Child and Adolescent Mental Health Care Services.

### 2.5 Data collection

Data collection was performed by the first author, a clinical psychologist who previously worked with children and young people, in January and February 2022. The interviews were performed at a location of each young person’s choice. Most interviews were conducted in the Youth Flexible ACT team locations, while some participants wanted to meet at school, in a park, or over the telephone. Eleven interviews were conducted face-to-face, while three were conducted over the phone. Considering that this group of young people can be difficult to recruit, they were encouraged to participate face-to-face, but they were also allowed to participate if they preferred a telephone interview. The young people spoke with the interviewer alone, except for one who wanted to bring a parent to the interview because of his psychotic symptoms.

A semi-structured interview guide with open-ended questions was designed with six main topics and corresponding subtopics. The material in this article primarily describes the young people’s responses to main topic 1, where the participants were asked how they experienced the follow-up and treatment provided by the Youth Flexible ACT team. The interview started with questions about age, living conditions, and reasons for seeking treatment. The questions asked included the following: ‘What has been helpful for you?’, ‘Have there been any challenges or negative aspects?’ and ‘Is Youth Flexible ACT different from previous assistance or treatment you have received, and if so, how?’. All participants were asked the same main questions to ensure consistency in the data collection. In addition, it was important to adapt the interview to the individual participant as some displayed difficulties in verbal skills, such as putting their thoughts into words, difficulty in articulation and reflecting verbally and in detail. Therefore, several prompts and questions such as “Could you say some more about that?” and “Could you give me any examples of that?” were used. These methods encouraged participants’ ability to respond to the questions and provide more detailed answers

The length of the interviews ranged from 25 to 60 minutes, with most lasting about 50 minutes. The interviews were audio-recorded, except for one in which the youth did not want to be recorded. Notes from this interview were included in the analysis. Participants were compensated with a gift card worth 500 NOK (approximately 47 USD).

### 2.6 Data analysis

We used qualitative content analysis to explore the experiences of the young people. This method is considered to be useful in analyses of experiences, reflections and attitudes of an individual or group (47). It includes descriptions of the manifest content as well as interpretations of the latent content or the underlying meaning (48). In terms of epistemology qualitative content analysis can be applied to different views of knowledge and the manifest descriptions can be said to contain phenomenological descriptions while the interpretive practice contains hermeneutic interpretations (49, 50).

Interviews were analysed following the principles of content analysis as described by Graneheim and Lundman (48). All interviews were transcribed verbatim to ensure reliability and the extraction of direct quotes. The analysis was performed in several steps. 1) All interview transcripts were read several times to become familiar with the material and to obtain a sense of the whole. 2) We identified meaning units in the text regarding the participants experiences of Youth Flexible ACT; these units consisted of words, sentences, or paragraphs related to each other through their content and context. 3) The meaning units were condensed; they were shortened while preserving the core meaning. 4) The condensed meaning units were abstracted by labelling them with codes. 5) The codes were compared for similarities and differences and sorted into five categories describing the content at a manifest level. Although the level of abstraction and interpretation varied (48), a low degree of interpretation is used on this level of analysis to stay close to the participants’ descriptions. 6) Two themes that unified the content in the categories at an interpretative/latent level were identified (48). To enhance trustworthiness the development of categories and themes was a back and forth process consisting of several discussions between the authors to reach consensus. The themes, categories and codes generated from this analysis are summarized in **Table 1**. Quotations are presented to illustrate the categories.

**Table 1:** The main themes, categories and codes derived fom the analysis of interview data.

Themes	Trusting and collaborative relationship			Organization matters	
**Categories**	Supported and accepted	Friend-like relationships	Focus on solutions	Help in different areas	Flexibility and availability
**Codes**	Being listened to Not given up on and given time Challenges Co-determination	Friend-like Closer in age	Solutions not problems Get things to do, not just talk	Multiple help and support Systemic family focus	Flexible Available

### 2.7 Ethical considerations

This study was approved by the Regional Committee for Medical Research Ethics (No. 600649). The processing of personal data was assessed by SIKT (REC South-East, 904802). This study was conducted in accordance with the Declaration of Helsinki.

The participants in this study can be considered ‘doubly vulnerable’ according to research ethics guidelines because they are both children and young people (age range, 12–25 years) and individuals with severe mental health problems (51). However, a risk of excluding groups and individuals from the study existed because they were considered too vulnerable. We needed this group’s valuable experiences and perspectives and instructed the staff to provide an invitation for participation to all the young people. Nevertheless, the staff can act as gatekeepers, for example, by excluding those with the most severe symptoms (52), although evidence has been accumulated regarding how the benefits of participating and positioning young people as agents in developing their services can facilitate youth empowerment (53). Instead of excluding young persons on the basis of possible severe mental health problems and symptoms, a safety net was established. They could have a follow-up talk with the staff from their team if needed. All participants were also under treatment from a Youth Flexible ACT team at the time they were interviewed, which ensured that they were cared for by qualified health professionals. They were informed that the interviews were confidential and that participation or non-participation in the project would not affect their treatment. The participants could withdraw from the study at any time without providing a reason. Several of the young people participating in the present study expressed that it was nice to be heard and that they benefited from it. None of the participants expressed negative experiences regarding participation.

## 3 Results

The following two main themes were identified: (1) trusting and collaborative relationships, and (2) organization matters. The main latent themes and manifest categories with their underlying content are presented in **Table 1**.

### 3.1 Trusting and collaborative relationship

#### 3.1.1 Supported and accepted

The participants expressed that they appreciated the opportunity to talk and be heard in Youth Flexible ACT. Being able to talk about their wishes, life and anything they wanted was highlighted. They said that they were heard and understood, felt more normal, accepted and human, and felt less alone with their thoughts. The quote below illustrates one of the participants’ descriptions of how she felt being heard, supported and accepted:

*But I feel here when I talk about something I think is bad, they agree that it’s bad in a way, and that’s made a very big difference. Because even though sometimes I can be a little bit sad in a way after a meeting, it’s more because I’ve got my feelings accepted and supported, and then I do not feel stupid for feeling that way, I feel more understood*.

The participants experienced that they were not judged for anything that they said. The staff created a trusting environment by validating their feelings and having a non-judgemental attitude. One participant said:

*That I can talk about what I want. That I sort of know that I won’t be judged for what I say because you can feel that*.

Several participants indicated that it was good to talk about their interests and do things that they were interested in with the staff. Interests such as listening to music and playing games together were the entry point for some participants to start conversations and build relationships and trust with the staff. Others said that it was nice to be able to choose to talk about their interests:

*Yes, I feel I’m listened to, I can use the time we’re together to talk about whatever I want. I think that’s fine because it’s not so much fun if I’m forced to talk about something, but if it’s something difficult to talk about, I can do that. I also like being able to come and talk about things I’m interested in*.

Being able to discuss absolutely anything and share one’s interests was viewed as a contrast to previous experiences with other health services, where the emphasis was on talking about one’s problems. One participant said:

*We can talk about absolutely everything, you know, and I don’t even need to talk about my problems every time. I’ve talked about piercings for example, like I want to get new piercings, but at the mental health clinic, it was my problems that I had to talk about*.

Some participants said that the staff did not give up on them. They described that the team continued to show up even if they were ill, cancelled appointments, were ambivalent or had limited energy to talk to the team. Other participants described that the staff listened to them no matter what, even if they ‘spoke too much’, talked negatively or had to explain themselves several times. To be given time was described as valuable. The teams did not focus on rushing things. Thus, the participants had time to establish a relationship with them and the shared focus on interests over a long time became an entry point to feeling secure and a different way of being together than discussing difficult topics. One participant shared the following perspective on how important the combination of interests and time was for gaining trust:

*I haven’t been able to delve into deep things yet because I want to make sure they (the team staff) are still there when I do. These moments with games help me. Even though we don’t talk very personally, it helps, just knowing that I have the opportunity to talk if I want, even if I maybe don’t, but just knowing that I can and that we can maybe play without me having to say anything but having the opportunity, it makes me feel good*.

Another topic several of the young people talked about was how the team challenged them in a caring and respectful way, which was described as both being challenged to do things and to talk about what is difficult and painful. The following quote from one participant illustrates this:

*They try to challenge me with challenges that I’m comfortable with because I try to challenge myself too, but if there’s something I think is way too difficult, they sort of let it go*.

Most participants mentioned that they experienced co-determination in the Youth Flexible ACT. They emphasized different areas of co-determination and talked about deciding what to do, talk about and get help for. A running theme was that they valued deciding where to meet the team, for example, at school, home or outside. A common experience of several participants was that they had independent wishes and needs that could conflict with the team’s and parents’ understanding, but experienced that their voice was heard and acted upon. For example, one participant said she was involved in deciding what kind of mapping was done:

*… it was a diagnosis that I thought I had, and then I sort of explained myself, but at first it was like ‘no, I (team staff) don’t really think so’ and now I’m going to be mapped and just to check, so it was a bit nice to have a hand in deciding*.

Some young people said that they had not received appropriate information about Youth Flexible ACT and that it was not adapted to them or sufficient for other services and people around them. They said that it was demanding to explain what Youth Flexible ACT was to various staff members at school, and this was experienced as a burden in addition to explaining one’s illness.

#### 3.1.2 Friend-like relationships

The relationship with the staff in the Youth Flexible ACT team, and especially with their contact person, was something that all the participants were concerned about. The relationships were said to be more personal in contrast to those in earlier treatment, which was experienced as consisting of more solemn meetings. Several participants expressed that they had close contact with the staff. For some, the contact person was their closest confidante, someone to whom they could tell everything. Many participants also described the employees as being friendly and someone who wanted to be with them. The following quote illustrates this:

*One thing I like is that he’s not just there to talk to you, he’s more like, what can I say, a friend in a way. It’s kind of easier that way because then I’m not just telling things to some person, I’m telling things to a person I actually trust, you see*.

Another young person had a similar experience with a genuine relationship. When asked what was particularly important, the young person replied:

*I think it’s that they actually care in a way, not just that they get their income and do their job*.

Some participants highlighted the importance of the staff’s age. One participant said that she did not want to talk to anyone who could have been her grandparent. Another young person stated that it was easier to talk to a person who was closer to them in age:

*I like it if I have a person who is a little closer to my age because then they understand a little more about my problems, we also have something more to talk about, such as games*.

Although the participants mainly reported positive relational experiences with the team staff, a few negative experiences emerged. No specific recurring themes were present, but such experiences included not feeling like they were being taken seriously, missing personal chemistry and inadequate emotional support. One young person who expressed that she was not taken seriously said:

*That he doesn’t take me seriously, very often. I feel that he starts talking about completely different things*.

#### 3.1.3 Focus on solutions

Participants said that they experienced employees in the team as solution-oriented. They emphasized that the staff proposed various solutions relevant to their challenges, including various school challenges, treatment methods, family conflicts, symptom management and challenges with friends. They provided tips and tricks, various tools and exposure tasks. Many participants highlighted that it was good to be provided specific things to do and not just have conversations:

*He* [team member] *kind of gives me more specific things to do, because, well me and maybe some others, we want something we can actually do, not just talk and then it’s the next session*.

Another young person said that solutions were provided if you had done something bad:

*If you have done something badly, they talk to you and find solutions, I think that is good*.

This quote from a young person also illustrates the experience of solutions and that the young person’s own wishes were in focus:

*I kind of feel that [the therapist] wants more of what I want. How I want, and if it’s a longer process, so be it, or he helps find solutions for it too, it’s not like if I say no to using medication or something he doesn’t bother, then he has no way to help me. Then he rather says ‘well, then we have to find some other solution’, if you understand*.

### 3.2 Organization matters

#### 3.2.1 Help in different areas

One area highlighted as a positive aspect by the participants was that the team assisted in multiple areas of their lives. Although few participants talked about this in detail, they described some aspects of how the Youth Flexible ACT was organized. Receiving different kinds of support in one place, including practical help, was described by some participants as a positive and distinctive feature compared with their previous experiences of mental health services. The participants mentioned that they could be accompanied to meetings, visit the library or listen to music with the team members. When asked if something in the Youth Flexible ACT differed from other treatments, one participant said:

*… that there are several people who work here, so you can get help with several things. I think it’s very nice, that everything is in one place. That you don’t have to go to several places*.

A few participants also expressed a desire for more activities to be provided by the team, such as conversation groups with other youth in the team and other activities to participate in, preferably alongside peers, such as cycling trips and excursions outside the city. Another theme the participants brought up was that the team consisted of multiple staff members, which provided them with various opportunities, such as making it easier to find a staff member they liked. Moreover, some participants mentioned that having a doctor on the team was beneficial.

Another aspect related to the holistic/systemic working of the team emphasized by several participants was the team’s engagement with the system surrounding the youth, where the family plays a central role. Some participants said that relational difficulties in the family were one reason they needed follow-up from the team. They further highlighted that help from the team had made their family members improve their communication and understand each other better. Some participants also described what the team did to succeed. For example, they described that their viewpoints were considered as important as those of the adults and parents. They felt that being able to control what was passed on to and talked about with the parents was important. They experienced that the team respected their boundaries. The following quote illustrates the importance of the team having a family focus:

*Yes, the conversation we’re having, I’d say it was maybe the first successful conversation between me and my mom, with, yes, with the Flexible ACT guys*.

The young person further described what was helpful:

*Maybe it’s with respect somehow, I feel like, that I know that my words are considered as important as my mother’s, that I know that they won’t be considered any different, even though she’s a grown lady, sort of, and I’m a little older kid, sort of. That I don’t get that nobody bothered to listen to me because I’m young, that sort of all the viewpoints are seen anyway*.

#### 3.2.2 Flexibility and availability

Another common theme among the participants concerned flexibility in the team, especially arena flexibility. They appreciated that they could decide where to meet the team, in or outside the office. The staff being able to come to school and their home was also mentioned as a positive aspect. Walking and talking together, getting a breath of fresh air during the conversation and going on car trips were also mentioned to be helpful to the participants. One participant said:

*It’s pretty cool, I don’t want to sit around the office. For example, it’s better to go for a walk, walk around and talk, I think that’s much better*.

Others said that they previously had been unable to attend treatment, but they could do so now because they did not have to come to the office at a fixed time:

*I think it’s really cool that* [the contact person] *can come and see me any time in the week, not at a fixed time, you know, a bit more flexible*.

The participants described that they perceived the team as accessible because the team office was located closer to their residence, and they could contact the employees via a mobile phone. They said that it was reassuring to have this option even if they knew that the staff did not work outside ordinary working hours and even if they did not get an answer immediately. Furthermore, the need for extended opening hours was highlighted. The following issues were raised by the participants: they mentioned that emergencies (thinking about taking their own lives) could arise after the team’s opening hours, and that it was challenging to receive treatment during school hours while dealing with school absenteeism. One young person mentioned that he often ended up having to prioritize school over appointments with Youth Flexible ACT:

*So, there can be times when it gets difficult if I’m having a bad day, it can be a bit challenging too, you know, then I have to prioritize either school or appointment with the team and then it’s a bit like, when I’m already struggling to get to school, there’s a bit of pressure from that side, so it usually ends up that I have to prioritize going to school*.

Others described having regular contact and appointments every week as positive. The participants also highlighted that they could get several appointments per week and at short notice:

*If things go a little harder, if I tell* [contact person]*, he sort of takes that into account and sets up more time to talk*.

Some participants described the advantages of having more employees in the team and appreciated that more employees could focus on the young person, which made it easier to get an appointment. A young person said:

*And if I suddenly just need an appointment one day, and then it may not be exactly my therapist who is available, but I get to talk to one. And I think that’s very good because I’ve talked to everyone here, I think*.

## 4 Discussion

To the best of our knowledge, the present study is the first to explore how young people experience follow-up and treatment provided by Youth Flexible ACT teams. Several previous studies on mental health services for young people have focused on documenting the views of parents and health-care professionals (37), despite their viewpoints being different from those of young people regarding what they perceived as good quality mental health care (36, 54). The young people in the present study provided rich and relevant information and had clear ideas about their care needs. Our findings challenge the notion that some groups are too vulnerable to participate in shaping the development of (their own) mental health services (55). Therefore, the present findings add to the scarce knowledge of what young people with severe mental health problems and complex needs want from their mental health services.The main findings from the analysis are summarized in the following themes: (1) trusting and collaborative relationships, and (2) organization matters.

The relationship with the team staff was important for the young people in the Youth Flexible ACT teams, consistent with previous findings (56, 57). The participants emphasized the importance of being listened to and the team staff focusing on their interests and resources, not only their problems. Most participants experienced a supportive and helpful relationship with the staff, but some experienced difficult situations when this had not been the case. Some participants experienced missing chemistry and not being taken seriously or given sufficient emotional support. Other studies on the same topics also found that young people want to be heard (58, 59) in a non-judgemental and attentive way (38, 60).

Continuity with no set time limit for the follow-up other than age (25 years) is one important element of the Youth Flexible ACT model and can prevent transitional youth from dropping through a known ‘care gap’ (61). The importance of continuous follow-up combined with a trusting and collaborative relationship was illustrated by one important finding in this study: the participants ‘tested’ (in secret) whether they could trust the staff before potentially sharing difficult experiences. A way of ‘testing’ could be by talking about things other than their difficulties for a long time or focusing on activities, to check whether the staff continued the relationship. Our findings support those of Stige et al. (62), who interviewed young people (age range, 15–19 years) about their experiences with mental health care. They found that the young people hid their true feelings and secretly tested therapists to check whether they cared. This finding is consistent with the description of youth as ‘the deceptive age’, where young people give misleading signals about their needs (63). This could be an explanation regarding why young people appear unmotivated in treatment settings when the services have not met their need for trust-building over a sufficiently long time. Our findings suggest that it is important for this target group to have the possibility of a long-term follow-up by staff who are curious about their behaviour, but do not delve rapidly into difficult topics, and to have their interests and meaningful activities given significant time and space during meetings. Although the Youth Flexible ACT teams were new in Norway, they worked differently from ordinary services by providing this kind of follow-up, which may indicate that the staff is working with a recovery-oriented approach (64), a core principle of the Youth Flexible ACT model. This way of organizing mental health services enables individually tailored services, and sufficient time can be a key element for the staff to be able to ‘pass the test’ of being trustworthy to young people.

The participants wanted a more personal relationship with the staff who were genuine, informal, less hierarchical and friend-like as opposed to them ‘just doing a job for a pay check’, and said that they experienced this with the team. Expressing a desire for this kind of relationship with the staff is consistent with previous findings on this topic (65–68). The present findings are similar to those reported by Ungar et al. (69) about risk and resilience profiles and boundaries in the therapeutic relationship. Ungar et al. (69) suggested that young people at higher levels of risk exposure, which would be the case for several in the target group of Youth Flexible ACT (32), prefer and need looser professional boundaries (a real relationship) (70) in contrast to those who have greater access to resources such as social support, where looser professional boundaries can potentially be disruptive. A dichotomy between personal connectedness and a professional role, and between loose and strict boundaries (71), can make it challenging for the staff in mental health services to establish friend-like relationships similar to what the participants preferred. The success of the Youth Flexible ACT team in building a therapeutic alliance can be explained in part by the Youth Flexible ACT model framework, which provides opportunities for the staff to step out of their traditional professional roles and settings and work in accordance with recovery values. Moreover, the staff have the opportunity to be more in the young people’s arena, meeting them where they prefer and in their community.

The participants wanted both self-decision and guidance and support from staff. This may be an expression of how they managed to balance the natural developmental conflict of autonomy and agency experienced by young people (63, 66). This key developmental task can make them vulnerable to hierarchy and power differentials, requiring the staff to balance power by simultaneously listening, offering solutions and providing therapeutic interventions (72). The finding in the present study that the attachment needs of young people were being met suggests that the staff were adept at providing developmentally appropriate care in this area and facilitating the crucial development process of gradually becoming more independent persons (63).

The young people in our study described much of the same themes as those in other studies on relational aspects (38, 59, 73–75). The majority also described being highly satisfied with their relationship with the staff. Interestingly, the team staff were primarily recruited from traditional services, and all the young people also had experiences with these services. Basically, the same staff and young people were meeting in a new setting in the Youth Flexible ACT framework, where the young people described being met differently and as they desired. This finding may indicate that the Youth Flexible ACT model enabled the staff to provide the follow-up and treatment that this group of young people both wanted and needed.

Receiving flexible and accessible help from Youth Flexible ACT in multiple areas was appreciated by the study participants. To our knowledge, research on organizational aspects is more limited than that on relational aspects (9). Muir et al. (76) highlighted this aspect and reported that young people in the mental health service Headspace appreciated the inclusion of physical health services and support across a broad range of areas. Discontinuity, with shifts between services, has been shown to be experienced as disturbing and unhelpful by young people in several studies (75). The Youth Flexible ACT model is designed to meet these barriers by providing integrated, holistic and continuous care. The participants described the advantages of follow-up, from different professionals and over time, in several areas of their lives. However, similar to findings from other studies, the present participants also requested extended opening hours that could accommodate school participation (59) and team availability during acute crises (60).

Our finding on what young people find as helpful systemic work with family involvement, for example, respect for boundaries and the young people being heard as equals to their parents, is consistent with those in previous studies. However, other studies have also reported that young people have more negative experiences and feel that involving parents is not helpful (37, 59, 73, 77). The way the Youth Flexible ACT teams are organized with a holistic focus, no time limit and multidisciplinary staff, preferably including a family therapist, appears to facilitate systemic work. One aspect of this organization that can promote the ability of the staff to provide appropriate help is the possibility of simultaneously offering individual and family treatment.

The participants deeply valued the assertive outreach and arena-flexible approach of the teams, which provides young people who cannot visit an office, crucial opportunities to receive treatment and more ways of building relationships with the staff than traditional office-based conversations. Our findings are consistent with those of Plaistow et al. (2014), who conducted a review of 22 studies on young people’s experiences with mental health services in the UK and found that accessibility was the most prevalent theme, including young people’s desire for an outreach approach and services being accessible in different locations, such as at school, and favoured home-based treatment over hospital admission.

Continuous follow-up during fluctuating mental health conditions and knowing that they were not given up on were experienced as being assuring by the participants in the present study. This finding is in agreement with the team’s mandate to provide more intensive support when needed and less frequent follow-up (individual case management) during better periods, thereby providing continuity in support. Gibson et al. (73) indicated that a ‘drop-in/drop-out’ engagement process can be characteristic of adolescents and that ‘services which allow flexible routes back into re-engagement, after ‘drop-out’ may be useful to young people’ [(73): p.1064]. Another study also described the dynamics of service use for a group of young adults to be characterized by complicated stops and starts (78). This finding is in agreement with those related to the developmental period, with shifting developmental tasks to accomplish (79), and high stress and emotional fluctuations being more common for some young people (80). Most importantly, it is a period of exploration and discovery (81) where the Youth Flexible ACT team’s flexible organization appears to be appropriate and supportive of the target group of young people.

## 5 Limitations and strengths

One strength of the present study is the rich data gathered in the interviews. The study comprises firsthand information from 14 young people in the teams, constituting a large proportion of the total 55 young people across the three teams. Both males and females participated. Even though the 14 participants struggled with different problems, they emphasized several of the same aspects of the Youth Flexible ACT model and provided rich and informal information. The participants had various psychological and social challenges. Some also had a significant symptom load at the time of the interviews. Therefore, they appear to represent diversity and not a selected group of the best-functioning young people.

The teams had been operating for approximately 2 years at the time of the interviews. They were initiated when limited knowledge about the Youth Flexible ACT model was available in Norway and no model handbook had been developed in Norwegian. Therefore, the team’s degree of model adherence is unclear. The finding that young people emphasized several components in the model may indicate that the staff predominantly followed the Youth Flexible ACT model description.

Youth Flexible ACT in Norway is intended for young people aged 12–25 years. The participants in this study were aged 15–19 years. Including younger and older participants might have provided other perspectives and highlighted other developmental challenges.

## 6 Conclusion

The results of this study point to the well-known key role of relationships, showing that a trusting and collaborative relationship between young people and the staff is experienced as crucial. The findings also add knowledge about the importance of service organization, implying that the possibility of achieving a fruitful relationship and providing appropriate treatment also lies in how the service is organized. Staff skills can be better utilized by providing supportive frameworks for health services. A comprehensive service model, including integrated, flexible and multifaceted follow-up, is needed to meet the needs of this group of young people. Meeting young people in a youth-friendly and developmentally sensitive manner, with attention to autonomy, resources, interests, solutions and context, should be considered a guiding principle when working with young people and further developing the Youth Flexible ACT model.

Further research on Youth Flexible ACT user experiences is needed to make the service more user-oriented. Exploring how to adapt services in better accordance with the needs of young people at different developmental stages and those with concurrent substance abuse and mental health issues could represent additional important research areas.

## Data Availability

The datasets presented in this article are not readily available to protect confidentiality. Requests to access the datasets should be directed to marthe.johansen@inn.no.
